# Supplementary material for: Identification of Survival-Specific Genes in Clear Cell Renal Cell Carcinoma Using a Customized Next-Generation Sequencing Gene Panel
Source: J Pers Med. 2022 Jan 14;12(1):113. doi: 10.3390/jpm12010113 (PMC8778284; doi:10.3390/jpm12010113)
Supplement: Supplementary file 1 [file jpm-12-00113-s001.zip › jpm-1488575-supplementary.pdf]

Journal of
Personalized
Medicine

Supplementary Materials

Table S1. (a). The performance of feature selection method_Information Gain (IG) depending on the weights and classifiers.

Information Gain	Classification Algorithms, Accuracy (%)		
**Weight (Gene Number)**	**Naïve Bayes**	**K-NN (K = 3)**	**SVM**
≥0.015 (17)	79.54	**72.32**	78.34
≥0.011 (78)	92.31	68.98	80.98
≥0.008 (95)	92.55	68.48	81.21
≥0.005 (537)	**99.04**	66.31	**83.86**

The highest values are bolded. K-NN, K-Nearest Neighbor; K, number of nearest neighbors; SVM, Support Vector Machine.

Table S1. (b). The performance of feature selection method_Chi-squared depending on the sample sizes and classifiers.

Chi-squared	Classification Algorithms, Accuracy (%)		
**Sample size (Gene Number)**	**Naïve Bayes**	**K-NN (K = 3)**	**SVM**
≥7 (20)	79.54	**72.56**	77.63
≥5 (85)	91.59	68.48	80.73
≥3 (461)	86.6	66.07	81.21
≥2 (621)	**97.37**	66.07	**83.61**

The highest values are bolded. K-NN, K-Nearest Neighbor; K, number of nearest neighbors; SVM, Support Vector Machine.

Table S1. (c). The performance of feature selection method_MRMR depending on the numbers of relevant genes and classifiers.

MRMR	Classification Algorithms, Accuracy (%)		
**Number of Relevant Genes**	**Naïve Bayes**	**K-NN (K = 3)**	**SVM**
Top 10	42.55	68.25	68.76
Top 20	76.67	**72.58**	72.84
Top 30	80.75	72.56	77.86
Top 100	**93.49**	67.27	**83.39**

The highest values are bolded. MRMR, Minimum Redundancy-Maximum Relevance; K-NN, K-Nearest Neighbor; K, number of nearest neighbors; SVM, Support Vector Machine.

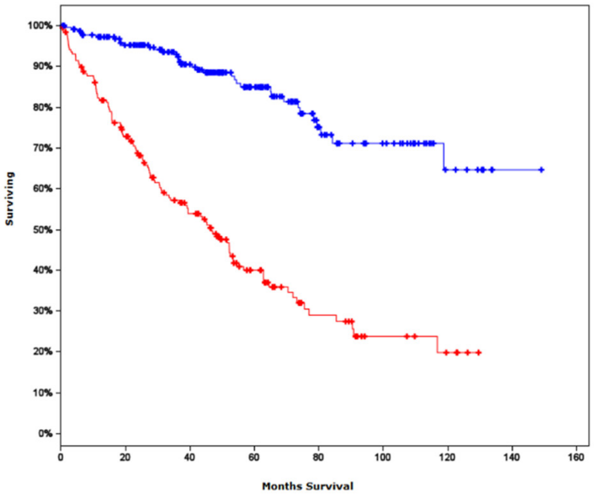

▷ **Information Gain (Survival-related Top 100 genes)**

- p − value: <0.001

	Total	Decease	Median Survival (Months)	Decease (%)
Mutation Positive	190	112	46.55	58.9
Mutation Negative	227	35	NA	15.4
	37	77		43.5

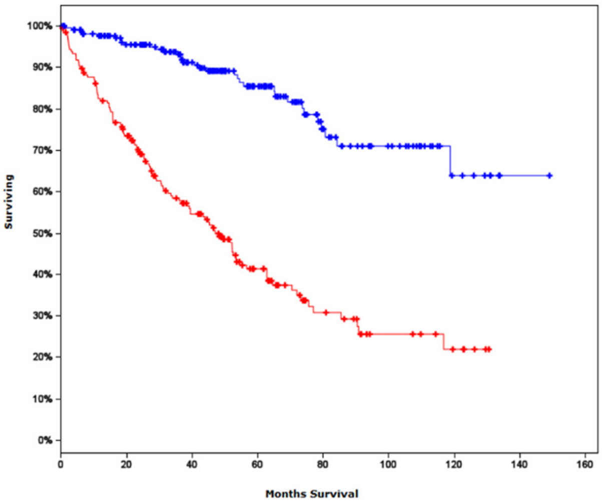

▷ **Chi-square (Survival-related Top 100 genes)**

- p − value: <0.001

	Total	Decease	Median Survival (Months)	Decease (%)
Mutation Positive	198	114	47.04	57.6
Mutation Negative	219	33	NA	15.1
	21	88		42.5

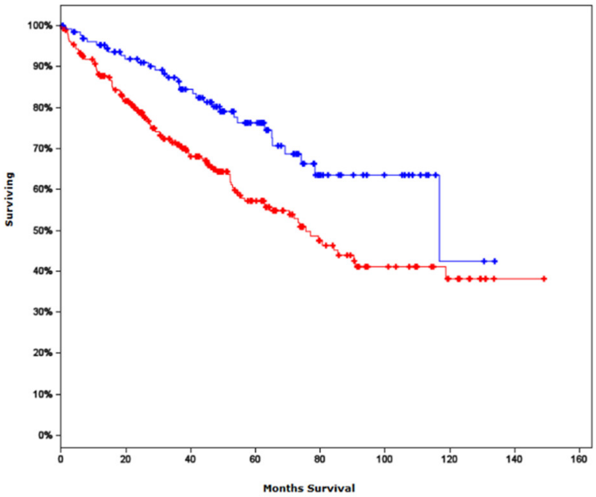

▷ **MRMR (Survival-related Top 100 genes)**

- p − value: <0.00112

	Total	Decease	Median Survival (Months)	Decease (%)
Mutation Positive	286	115	75.53	40.2
Mutation Negative	131	32	116.75	24.4
	155	83	41.22	15.8

Figure S1. Survival curves of top 100 genes by three feature selection methods (Information Gain, Chi-squared test, and MRMR). NA, not available; MRMR, Minimum Redundancy-Maximum Relevance.

Table S2. A total of 123 survival-specific mutations selected in TCGA-KIRC using three feature selection methods (Information Gain, Chi-squared test, and MRMR).

Feature Variable (Clinicopathologic Factors)	Clinicopathologic Factor-Related Mutant Gene Number	Survival-Specific Mutant Gene Number
Age	109	15
Gender	68	9
Stage	76	32
Recurrence	40	38
Metastasis	58	30
Survival	42	34
Total (deduplicated)	195	123

Table S3. A total of 21 of top-ranked mutant genes above 5% in TCGA-KIRC.

Gene	Mutation Frequency (%)	Mutation Number	Sample Number
VHL	49.9	241	225
PBRM1	30.6	141	138
MUC4	17.1	129	77
TTN	15.5	84	70
MUC16	12.9	83	58
SETD2	11.3	58	51
BAP1	8.9	42	40
MTOR	7.1	33	32
FBN2	6.7	39	30
XIRP2	6.2	30	28
KDM5C	6	27	27
DNAH3	6	33	27
HMCN1	5.8	26	26
ARID1A	5.5	28	25
TSHZ3	5.5	32	25
ARAP3	5.5	38	25
SSH2	5.3	28	24
VWF	5.1	35	23
AKAP9	5.1	29	23
SPEN	5.1	25	23
NAV3	5.1	26	23

Table S4. A total of 14 genes for other solid tumors.

Gene	Mutation Frequency (%)	Mutation Number	Sample Number
BRAF	0.4	3	2
BRCA1	1.1	6	5
BRCA2	1.6	7	7
EGFR	2	9	9
ALK	0.9	4	4
ERBB2 (HER2)	0.9	4	4
IDH1	0.4	2	2
IDH2	1.1	5	5
MYCN	0.2	1	1
MYC	NA	NA	NA
KIT	NA	NA	NA
KRAS	0.2	1	1
PDGFRA	1.1	6	5
NRAS	NA	NA	NA

NA, not available.

Table S5. The Pearson's correlation test revealed that the presence of a mutation in *CARD6* was inversely related to the period of OS (Patient P10: 14 months, the shortest).

Characteristics	***CARD6* Mutation**				
Overall Survival, Month	Patient *n* (%)	Yes	No	*r* −0.441	*p*-Value 0.04
10–20	3 (14%)	1	2		
21–30	1 (4%)	0	1		
31–40	12 (54%)	0	12		
41–50	6 (27%)	0	6		

Table S6. The Pearson's correlation test showed that the presence of the *BAP1* mutation was moderately negatively related to the period of DFS (Patient P8: 28 months and alive, Patient P12: 0 month and alive, Patient P16: 1 month and dead, and Patient P22:1 month and dead).

Characteristics	***BAP1* Mutation**				
Disease Free Survival, Month	Patient *n* (%)	Yes	No	*r* −0.465	*p*-Value 0.029
0–9	5 (23%)	3	2		
10–20	4 (18%)	0	4		
21–30	3 (14%)	1	2		
31–40	8 (36%)	0	8		
41–50	2 (9%)	0	2		

Table S7. The Pearson's correlation test revealed that the presence of a mutation in *SECISBP2L* was rigorously related to the spread of cancer cells to the lymph nodes and positively related to death (Patient P11: positive and dead).

Characteristics	*SECISBP2L* Mutation				
N-stage (Lymph Nodes)	Patient *n* (%)	Yes	No	*r*<br>1	*p*-Value<br>2.20e–16
Positive (1)	1 (5%)	1	0		
Negative (0)	21 (95%)	0	21		
Death				0.463	0.03
Dead (1)	4 (18%)	1	3		
Alive (0)	18 (82%)	0	18		

Table S8. The Pearson's correlation test revealed that the presence of a mutation in *SETD2* was moderately related to nuclear grade (Patient P3: grade 4, Patient P5: grade 3, Patient P8: grade 3, Patient P11: grade 4, Patient P12: grade 4, Patient P18: grade 2, Patient P20: grade 3, Patient P21: grade 3 and Patient P22: grade 4).

Characteristics	*SETD2* Mutation				
Nuclear Grade	Patient *n* (%)	Yes	No	*r*<br>0.451	*p*-Value<br>0.035
1	0	0	0		
2	6 (27%)	1	5		
3	11 (50%)	4	7		
4	5 (23%)	4	1		

Table S9. The Pearson's correlation test revealed that the presence of a mutation in *NLRP2* was strongly associated with sarcomatoid component (Patient P5: positive).

Characteristics	*NLRP2* Mutation				
Sarcomatoid Component	Patient *n* (%)	Yes	No	*r*<br>0.69	*p*-Value<br>0.0004
Positive (1)	2 (10%)	1	1		
Negative (0)	20 (90%)	0	20		

Table S10. Although mutation in *OBSCN* and sex were moderately associated, we noticed that both patients had *OBSCN* mutation were female (Patient P6 and Patient P12) in this case. It is also observed that Patient P12 had sarcomatoid component (Patient P12: positive).

Characteristics	*OBSCN* Mutation				
Sex	Patient *n* (%)	Yes	No	*r*<br>0.462	*p*-Value<br>0.03
Male (0)	15 (68%)	0	15		
Female (1)	7 (32%)	2	5		
Sarcomatoid Component				0.474	0.026
Positive (1)	2 (10%)	1	1		
Negative (0)	20 (90%)	1	19		

Table S11. The Pearson's correlation test revealed that the presence of a mutation in *USP40* was strongly associated with having sarcomatoid component (Patient P5: positive).

Characteristics	*USP40* Mutation				
Sarcomatoid Component	Patient *n* (%)	Yes	No	*r*<br>0.69	*p*-Value<br>0.00038
Positive (1)	2 (10%)	1	1		
Negative (0)	20 (90%)	0	20		

Table S12. The Pearson's correlation test revealed that the presence of a mutation in *AKAP9* was strongly associated with having sarcomatoid component (Patient P5: positive).

Characteristics	*AKAP9* Mutation				
Sarcomatoid Component	Patient *n* (%)	Yes	No	*r*<br>0.69	*p*-Value<br>0.00038
Positive (1)	2 (10%)	1	1		
Negative (0)	20 (90%)	0	20		

Table S13. The Pearson's correlation test revealed that the presence of a mutation in *KDM5C* was strongly related to the fact that cancer tissue had spread to the nearby lymph nodes (P11: positive).

Characteristics	*KDM5C* Mutation				
N-stage (Lymph Nodes)	Patient *n* (%)	Yes	No	*r*<br>0.69	*p*-Value<br>0.00038
Positive (1)	1 (5%)	1	0		
Negative (0)	21 (95%)	1	20		

Table S14. The Pearson's correlation test revealed that *ADAMTS10* mutation was moderately associated with tumor size. Patient P18 had *ADAMTS10* mutation showed the largest tumor size (14.5cm) among 22 patients (size of tumors without mutation- mean: 6.2 cm, median: 5.5 cm).

Characteristics	*ADAMTS10* Mutation				
Tumor Size, cm	Patient *n* (%)	Yes	No	*r*<br>0.585	*p*-Value<br>0.004
≤7.0	14 (63%)	0	16		
>7.0	8 (37%)	1	5		

Table S15. The Pearson's correlation test revealed that *ARID1A* mutation is moderately associated with tumor size. Patient P18 had *ARID1A* mutation showed the largest tumor size (14.5 cm) among 22 patients.

Characteristics	*ARID1A* Mutation				
Tumor Size, cm	Patient *n* (%)	Yes	No	*r*<br>0.585	*p*-Value<br>0.004
≤7.0	14 (63%)	0	16		
>7.0	8 (37%)	1	5		
